# *Cornu Cervi Pantotrichum* Supplementation Improves Exercise Performance and Protects against Physical Fatigue in Mice

**DOI:** 10.3390/molecules19044669

**Published:** 2014-04-15

**Authors:** Chi-Chang Huang, Yi-Ming Chen, Nai-Wen Kan, Hui-Ling Chao, Chin-Shan Ho, Mei-Chich Hsu

**Affiliations:** 1 Graduate Institute of Sports Science, College of Exercise and Health Sciences, National Taiwan Sport University, Taoyuan 33301, Taiwan; 2 Graduate Institute of Athletics and Coaching Science, College of Sports and Athletics, National Taiwan Sport University, Taoyuan 33301, Taiwan; 3 Center for Liberal Arts, Taipei Medical University, Taipei 11031, Taiwan; 4 Department of Sports Medicine, Kaohsiung Medical University, Kaohsiung 80708, Taiwan

**Keywords:** deer antler, grip strength, antifatigue

## Abstract

*Cornu cervi pantotrichum* (CCP) is a well-known yang-invigorating agent used in traditional Chinese medicine that can nourish the blood, tonify qi, and invigorate bones and tendons with multifunctional bioactivities. However, evidence on the effects of CCP on exercise performance and physical fatigue is limited. We evaluated the potential beneficial effects of ethanolic extract from CCP on ergogenic and antifatigue functions following a physiological challenge. Male ICR mice from four groups (*n* = 8 per group) were orally administered CCP for 14 days at 0, 2054, and 4108 mg/kg/day, and were respectively designated as the vehicle, CCP-1X, and CCP-2X groups. The physical performance and antifatigue function were evaluated using forelimb grip strength and exhaustive swimming time as well as serum levels of lactate, ammonia, glucose, and creatine kinase after a 15-min swimming exercise. The results indicated that CCP-1X supplementation significantly improved grip strength; reduced fatigue-associated biochemical indices, including lactate and ammonia levels; and ameliorated skeletal muscle injury induced by acute exercise challenge. A trend analysis revealed that CCP supplementation significantly increased grip strength and dose-dependently reduced serum alkaline phosphatase, uric acid, triacylglycerol, and glucose levels in healthy mice. Therefore, CCP is a potential agent with an antifatigue pharmacological effect.

## 1. Introduction

*Cornu cervi pantotrichum* (CCP), or deer antler, is a well-known traditional Chinese medicine (TCM) recorded in the *Compendium of Materia Medica* (*Bencao Gangmu*) by Li Shi-Zhen approximately 500 years ago. CCP is believed to invigorate the spleen, strengthen bone and skeletal muscles, and promote blood flow [1]. The main nutrients and chemical constituents in CCP are proteins, amino acids, minerals, lipids, polypeptides, polysaccharides, phospholipids, and trace elements including Ca, P, K, Al, Zn, Cu, and Fe [1,2]. CCP has been extensively used in TCM as an antioxidant and for improving sexual function, postponing fatigue, and treating various disorders such as osteoporosis [1,3,4]. In addition, both *in vitro* and *in vivo* pharmacological studies have demonstrated that CCP exhibits antithrombotic [5], endocrine anticoagulation and antithrombotic [6,7], antiaging [8], immunomodulatory, anticancer, and antiinflammatory properties [1]. However, relatively few studies have directly addressed the potential ergogenic or antifatigue functions of CCP.

Fatigue is defined as physical and/or mental weariness resulting in negative effects on exercise intensity, work performance, family life, and social relationships [9]. Physical fatigue can be accompanied by deterioration in functional performance [10]. At least two mechanisms can explain the occurrence of physical fatigue: oxidative stress and energy exhaustion [11]. Exhaustive or intensive exercise can lead to the accumulation of excess reactive free radicals, resulting in tissue damage. Exhaustion theory suggests that energy source depletion and excess metabolite accumulation can lead to fatigue [12,13]. In this study, we evaluated the potential ergogenic and antifatigue effects of CCP by using our previously established *in vivo* platform [14,15].

## 2. Results and Discussion

### 2.1. Body Weight and Other Metabolism-Related Organ Weights

Morphological data from each experimental group are summarized in Table 1. The initial and final body weights, food intake, and water consumption did not differ among the three treatment groups. In addition, the groups did not differ in absolute weights of the liver, muscle, heart, lung, kidney, testis, epididymal fat pad (EFP), and brown adipose tissue (BAT).

**Table 1:** General characteristics of the experimental groups.

Characteristic	Vehicle	CCP-1X	CCP-2X	Trend analysis
Initial BW (g)	30.1 ± 0.3	29.4 ± 0.4	30.5 ± 0.3	0.5798
Final BW (g)	33.8 ± 0.8	34.6 ± 0.6	34.7 ± 0.7	0.5583
Food intake (g/day)	6.91 ± 0.28	7.23 ± 0.22	7.29 ± 0.24	0.3765
Water intake (mL/day)	7.93 ± 0.14	7.51 ± 0.22	8.27 ± 0.25	0.4492
Liver (g)	1.86 ± 0.06	1.87 ± 0.02	1.81 ± 0.04	0.2206
Muscle (g)	0.34 ± 0.01	0.32 ± 0.01	0.35 ± 0.01	0.7708
Heart (g)	0.21 ± 0.00	0.21 ± 0.01	0.22 ± 0.00	0.5426
Lung (g)	0.32 ± 0.04	0.32 ± 0.04	0.35 ± 0.04	0.4720
Kidney (g)	0.57 ± 0.02	0.55 ± 0.01	0.58 ± 0.02	0.8445
Testis (g)	0.21 ± 0.01	0.23 ± 0.01	0.21 ± 0.00	0.2130
EFP (g)	0.65 ± 0.04	0.56 ± 0.05	0.58 ± 0.04	0.4589
BAT (g)	0.12 ± 0.01	0.12 ± 0.01	0.12 ± 0.01	0.0882

Data are mean ± SEM for *n* = 8 mice in each group. Muscle mass includes both gastrocnemius and soleus muscles in the back part of the lower legs. EFP: epididymal fat pad; BAT, brown adipose tissue.

### 2.2. Effect of CCP Supplementation on Forelimb Grip Strength and Exercise Performance in a Weight-Loaded Swimming Test

**Figure 1:**
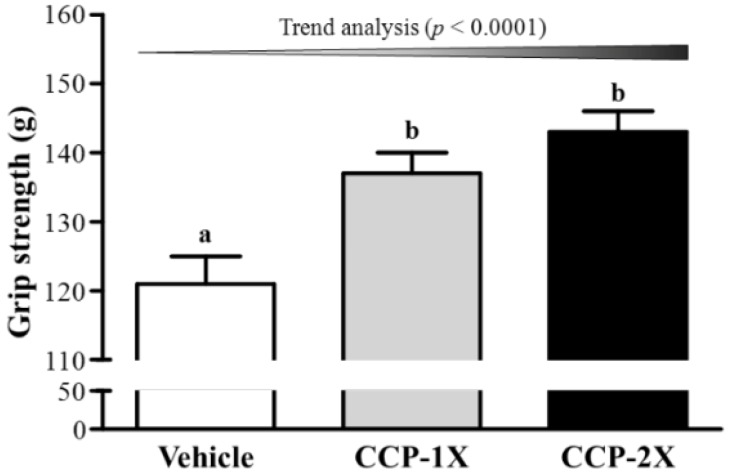
Effect of CCP supplementation on forelimb grip strength performance. Data are presented as the mean ± SEM of 8 mice in each group. Different letters (a, b) indicate a significant difference at *p* < 0.05 by one-way ANOVA.

As shown in Figure 1, the forelimb grip strength values in the vehicle, CCP-1X, and CCP-2X groups were 121, 137, and 143 g, respectively; the values of the CCP-1X and CCP-2X groups were significantly 1.13- (*p* = 0.0103) and 1.19-fold (*p* = 0.0008) higher than those of the vehicle group. In the trend analysis, absolute forelimb grip strength dose-dependently increased as the CCP dose (*p* < 0.0001) increased. A regulatory training program is required to increase grip strength [16]; however, the results indicated that CCP supplementation benefited grip strength even though the mice did not undergo a training intervention. Thus, short-term CCP treatment can benefit grip strength when no training intervention is implemented. Previous reports have shown that 7 to 21 days of supplementation with plant extracts or resveratrol or long-term supplementation with agents such as whey protein improves the grip strength of untrained and undertrained animals [16]. Thus, CCP, a substance of animal origin used in TCM, may be an alternative supplement for promoting body strength in a programmed training protocol. 

Exercise endurance is another variable used to evaluate exercise endurance performance. In our study, the exercise endurance levels of mice subjected to a swimming test and administered the vehicle, CCP-1X, and CCP-2X were 7.1 ± 0.7, 14.7 ± 6.2, and 6.8 ± 1.3 min, respectively. However, no significant differences in swimming times were observed among the vehicle and CCP treatment groups. One recent review reported that aqueous extract of deer antler base prolonged the endurance exercise time in untrained test animals [1]. However, a previous report showed that 10 weeks of elk velvet antler supplementation combined with training did not improve the rowing performance of male and female rowers [17]. Based on these results, we suggest that CCP, ethanolic extract of deer antler, improves body strength but not endurance performance in the absence of training. Therefore, further investigation is required to elucidate the effects of long-term CCP supplementation combined with exercise training on endurance performance. 

### 2.3. Effect of CCP Supplementation on Serum Lactate, Ammonia, Glucose, and Creatine Kinase Levels after Acute Exercise Challenge

**Figure 2:**
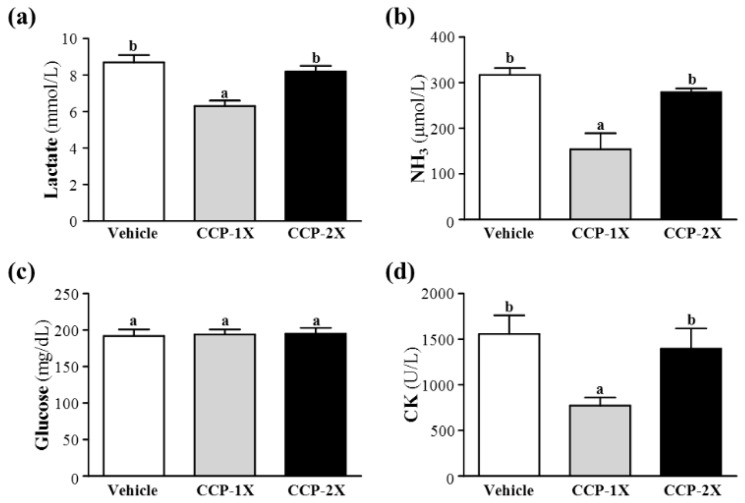
Effect of CCP supplementation on serum levels of lactate (**a**), ammonia (**b**), glucose (**c**), and CK (**d**) after a 15-min swim test without weight-loading. Data represent the mean ± SEM of 8 mice in each group. Columns with different letters (a, b) significantly difference, *p* < 0.05 by a one-way ANOVA.

Muscle fatigue after exercise can be evaluated using crucial biochemical indicators including lactate, ammonia, glucose, and creatine kinase (CK) after exercise [18]. During high-intensity exercise, muscles must obtain sufficient energy through anaerobic glycolysis, and a substantial amount of lactate is produced through glycolysis metabolism. The increased lactate level reduces the pH value, resulting in various biochemical and physiological side effects on glycolysis, phosphofructokinase, and muscular contractions caused by calcium ion release [19]. In the present study, respective lactate levels in the vehicle, CCP-1X, and CCP-2X groups were 8.7 ± 1.1, 6.3 ± 0.9, and 8.2 ± 0.9 mmol/L; only the lactate levels of the mice that received CCP-1X supplementation were significantly lower than those of mice that received the vehicle treatment (27.94% difference; *p* < 0.0001; Figure 2a).

Ammonia, a metabolite of proteins and amino acids, was linked to fatigue as early as 1922. The immediate source of ammonia produced from the deamination of AMP to inosine monophosphate through the purine nucleotide cycle is the greatest during intensive exercise when the rate of ATP use may exceed the rate of ATP production. Muscle fatigue is associated with the deamination of adenine nucleotides, and increased deamination of AMP coincides with decreases in phosphocreatine and pH values as well as failure of the contraction process. Peripheral and central fatigue levels are related to increased ammonia levels during exercise [20]. The serum ammonia levels in the vehicle, CCP-1X, and CCP-2X groups were 317 ± 47, 154 ± 112, and 279 ± 26 μmol/L, respectively; only the serum ammonia levels of mice that received CCP-1X supplementation were significantly lower than those of mice that received the vehicle treatment (51.26% difference; *p* = 0.0002; Figure 2b).

Glucose, a breakdown product of tissue glycogen, is released as a circulating substrate and used as energy after intense exercise [21]. During exercise, the plasma glucose level increases through the combined action of glucagon, epinephrine, norepinephrine, and cortisol. Although insulin enables glucose to enter a body cell, glucose declines during prolonged exercise. Exercise and muscle contractions increase glucose uptake in skeletal muscles through a mechanism that is independent of the insulin signaling pathway [22]. Figure 2c shows that the respective levels of serum glucose in the vehicle, CCP-1X, and CCP-2X groups were 192 ± 28, 194 ± 23, and 195 ± 24 mg/dL. No significant differences were observed in serum glucose levels among the vehicle and CCP treatment groups after a 15-min swim test.

Serum CK is a crucial clinical biomarker for various types of muscle damage, such as muscular dystrophy, severe muscle breakdown, myocardial infarction, autoimmune myositides, and acute renal failure. CK activity levels in the vehicle, CCP-1X, and CCP-2X groups were 1558 ± 643, 772 ± 277, and 1396 ± 704 U/L, respectively, and only CK levels in the CCP-1X group were significantly lower than those in the vehicle group (50.47% difference; *p* = 0.0121; Figure 2d). 

Thus, our results suggested that a daily recommended dose of CCP efficiently relieved fatigue-associated biochemical indices, including lactate and ammonia levels, and ameliorated skeletal muscle injury induced by acute exercise challenge. 

### 2.4. Effect of CCP Supplementation on Biochemical Analyses at the End of the Experiment

**Figure 3:**
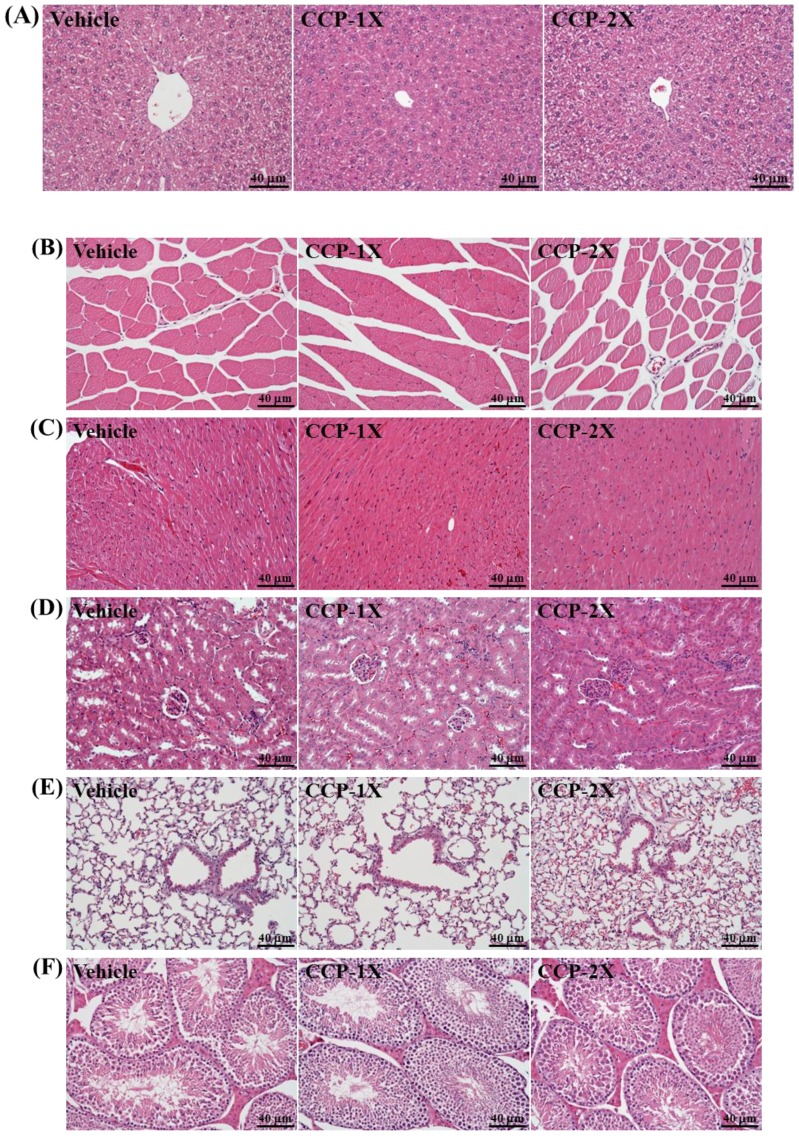
Effect of CCP supplementation on the morphology of liver (**A**), skeletal muscle (**B**), heart (**C**), kidney (**D**), lung (**E**), and testis (**F**). Specimens were photographed with a light microscope (Olympus BX51). (H&E stain, magnification: ×200, Scale bar, 40 μm).

In the present study, we observed the beneficial effects of a daily recommended dose of CCP supplementation on grip strength performance and fatigue-associated biochemical indices postexercise. Therefore, we examined whether administering CCP treatments for 14 days exerts negative effects on other biochemical markers in healthy mice. In a previous acute study, rats of both sexes were exposed to a dose of 2,000 mg/kg body weight. No mortality or other signs of toxicity were observed during 14 days of observation [23]. Furthermore, our histopathological examinations revealed that CCP supplementation for 14 days yielded no adverse effects in major organs such as the liver, skeletal muscle, heart, kidney, lung, and testis (Figure 3). We determined that the liver- and kidney-related biochemical parameters were nontoxic in CCP-treated mice (Table 2). Therefore, the dose of CCP supplementation used in this study was safe. Table 2 shows that the serum ALP, TC, TG, and glucose levels in the CCP-2X group were significantly 25.19% (*p* = 0.0094), 10.03% (*p* = 0.0465), 47.73% (*p* < 0.0001), and 12.39% (*p* = 0.0100), respectively, lower than those of the vehicle group. The trend analysis revealed that CCP supplementation dose-dependently reduced serum levels of ALP (*p* < 0.0001), UA (*p* = 0.0111), TG (*p* < 0.0001), and glucose (*p* = 0.0055). Our results were consistent with those of a previous report indicating that velvet antler supplementation significantly reduced serum ALP levels compared with those of a control group [4]. CCP supplementation produces no side effects *in vivo* and exhibits potential for application in measuring blood glucose, UA, and hyperlipidemia according to our *in vivo* data.

**Table 2:** Biochemical analysis of the CCP treatment groups at the end of the experiment.

Parameter	Vehicle	CCP-1X	CCP-2X	Trend analysis
AST (U/L)	78 ± 6	78 ± 10	72 ± 6	0.4773
ALT (U/L)	53 ± 4	44 ± 4^ a^	46 ± 2	0.1381
ALP (U/L)	444 ± 29^ b^	382 ± 35^ ab^	332 ± 15^ a^	<0.0001 (↓)
LDH (U/L)	396 ± 26	451 ± 37	428 ± 21	0.3274
CK (U/L)	245 ± 33	282 ± 90	187 ± 25	0.3077
Albumin (g/dL)	3.3 ± 0.1	3.4 ± 0.0	3.3 ± 0.1	1.0000
TP (g/dL)	5.6 ± 0.1	5.8 ± 0.1	5.6 ± 0.1	0.9163
BUN (mg/dL)	25.2 ± 0.9	25.2 ± 1.1	22.4 ± 0.9	0.0840
Creatinine (mg/dL)	0.30 ± 0.0	0.31 ± 0.02	0.33 ± 0.01	0.0600
UA (mg/dL)	1.16 ± 0.12	1.06 ± 0.13	0.89 ± 0.05	0.0111 (↓)
TC (mg/dL)	160 ± 4^ b^	168 ± 5^ b^	144 ± 7^ a^	0.0884
TG (mg/dL)	162 ± 13^ b^	137 ± 10^ b^	84 ± 8^ a^	<0.0001 (↓)
Glucose (mg/dL)	201 ± 5^ b^	186 ± 6^ ab^	176 ± 7^ a^	0.0055 (↓)

Values are mean ± SEM for n = 8 mice per group. Values in the same row with different superscript letters (a, b) differ significantly, *p* < 0.05 by one-way ANOVA. AST, aspartate aminotransferase (AST); alanine aminotransferase (ALT); alkaline phosphatase (ALP); creatine kinase (CK); lactate dehydrogenase (LDH); total protein (TP); blood urea nitrogen (BUN); uric acid (UA); total cholesterol (TC); triacylglycerol (TG).

### 2.5. Effect of CCP Supplementation on Histological Examinations at the End of the Experiment

Figure 3 shows that the three groups did not differ according to histological observations of the liver, muscle, heart, kidney, lung, and testis.

## 3. Experimental

### 3.1. Materials, Animals, and Experiment Design

**Figure 4:**
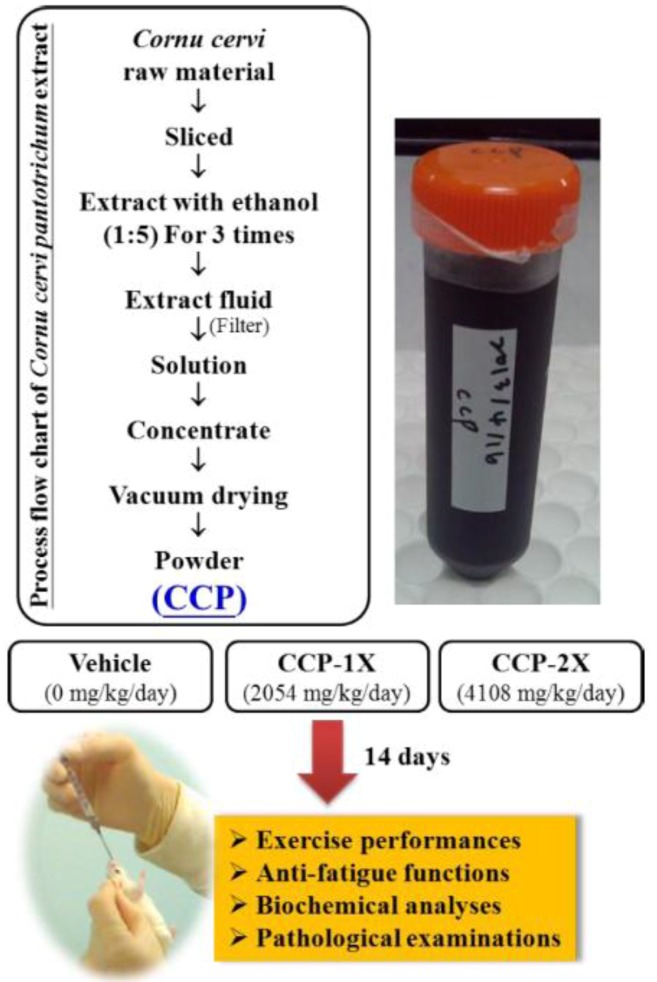
Protocol for preparation of ethanolic extract from *Cornu cervi pantotrichum* and experimental design of the study.

CCP ethanolic extract powder was purchased from International Total Solution (Taiwan), Inc. (Taipei City, Taiwan). Figure 4 shows the protocol used for preparing CCP and the experimental design of the study. Male ICR-strain mice (6 weeks old) with a specific pathogen-free condition were purchased from BioLASCO (Yi-Lan, Taiwan). All animals were provided with a standard laboratory diet (No. 5001; PMI Nutrition International, Brentwood, MO, USA) and distilled water ad libitum, and housed under a 12-h light/12-h dark cycle at room temperature (22 ± 1 °C) and 50%–60% humidity. The Institutional Animal Care and Use Committee (IACUC) of National Taiwan Sport University evaluated all animal experiments, and this study conformed to the guidelines of protocol IACUC-10109 and was approved by the IACUC ethics committee.

The recommended dose of CCP for humans is approximately 10 g with a normal diet. The CCP dose (2054 mg/kg) administered to mice in this study was converted from a human-equivalent dose (HED) based on body surface area by using the following formula provided by the U.S. Food and Drug Administration: Assuming a human weight of 60 kg, the HED for 10 (g) ⌯ 60 (kg) = 0.167 × 12.3 = a mouse dose of 2054 mg/kg. The conversion coefficient 12.3 was used to account for differences in body surface area between a mouse and a human, as described in our recent study [16]. 

After a 1-week adaptation period, all mice were randomly assigned to three groups (*n* = 8 per group) for CCP treatment: (1) the vehicle treatment; (2) 2,054 mg/kg of CCP (CCP-1X), or (3) 4108 mg/kg of CCP (CCP-2X). The vehicle group received the same volume of solution equivalent to the individual body weight. Both the vehicle and CCP were administered orally to each animal for 14 days.

### 3.2. Forelimb Grip Strength

A low-force testing system (Model-RX-5, Aikoh Engineering, Nagoya, Japan) was used to measure the forelimb grip strength of mice undergoing vehicle or CCP treatments. The amount of tensile force exerted by each mouse was measured using a force transducer equipped with a metal bar (2 mm in diameter and 7.5 cm in length). The detailed procedures have been described in our previous reports [14,15]. The test of forelimb grip strength was performed after the vehicle or CCP was administered consecutively for 14 days and 1 h after the last treatment. The maximal force (in grams) recorded using this low-force system was used as the grip strength.

### 3.3. Swimming Exercise Performance Test

The swim-to-exhaustion exercise test involved applying constant loads corresponding to 5% of the body weight of each mouse to evaluate endurance, as described in our previous report [24]. The endurance of each mouse was recorded as the time from the beginning of the test to exhaustion, which was determined by observing loss of coordinated movements and failure to return to the surface within 7 s.

### 3.4. Determination of Fatigue-Associated Biochemical Indices

The effects of CCP supplementation on fatigue-associated biochemical variables were evaluated postexercise according to the procedure described in our previous reports [14,15,24]. At 1 h after CCP supplementation, all animals underwent a 15-min swim test without weight loading, after which a blood sample was immediately collected and centrifuged at 1,500 *×g* and 4 °C for 10 min to prepare the serum sample. Serum lactate, ammonia, glucose, and CK levels were quantified using an autoanalyzer (Hitachi 7060, Hitachi, Tokyo, Japan). 

### 3.5. Blood Biochemical Assessments and Histological Staining of Tissues

At the end of the experimental period, all mice were finally sacrificed by asphyxiating them with 95% CO_2_, and blood was immediately collected at rest. Serum was collected by conducting centrifugation, and the clinical biochemical variables were measured using an autoanalyzer, as shown in Table 2 (Hitachi 7060). Various tissues were collected and fixed in 10% formalin after the mice were sacrificed. The tissues were cut transversely or longitudinally to obtain ventricular sections or 4-chamber cross-sections, respectively. The tissues were subsequently embedded in paraffin and cut into 4-μm thick slices for morphological and pathological evaluations. The tissue sections were stained with hematoxylin and eosin (H&E) and examined using a light microscope equipped with a CCD camera (BX-51, Olympus, Tokyo, Japan) by a veterinary pathologist.

### 3.6. Statistical Analysis

All data are expressed as the mean ± SEM. Statistical differences among groups were analyzed by conducting a one-way analysis of variance (ANOVA), and the Cochran-Armitage test was used to perform a dose-effect trend analysis by employing SAS Version 9.0 (SAS Institute, Cary, NC, USA). A *p* value < 0.05 indicated statistical significance.

## 4. Conclusions

Our result suggested that a daily recommended dose of CCP administered in the absence of training significantly improved grip strength; efficiently relieved fatigue-associated biochemical indices, including lactate and ammonia levels; and ameliorated skeletal muscle injury induced by acute exercise challenge, but had no effect on endurance performance. Therefore, further investigation is required to elucidate the effects of CCP supplementation combined with exercise training on exercise performance and physical fatigue. In conclusion, our study provides experiment-based evidence to support traditional claims regarding the antifatigue effect of CCP supplementation and suggests that CCP can be used as an ergogenic and antifatigue agent.
